# Comparative Evaluation of Perfusion Index With Lactate and Base Deficit for Assessing Resuscitation Response in Traumatic Shock Patients Not Requiring Blood Transfusion in the Emergency Department

**DOI:** 10.7759/cureus.113061

**Published:** 2026-07-20

**Authors:** Aghnay Kalathingal, Devendra Prasad KJ, Rajesh K, Krishnamurthy DGSR

**Affiliations:** 1 Emergency Medicine, Sri Devaraj Urs Medical College, Kolar, IND

**Keywords:** base excess, general trauma surgery, hemorrhagic, lactic acid, perfusion index, photoplethysmography, resuscitation, shock, spo2- saturation of peripheral oxygen, traumatic shock

## Abstract

Introduction

Traumatic shock is a life-threatening condition characterized by inadequate tissue perfusion following severe injury. Early recognition of impaired perfusion and monitoring of the response to resuscitation are essential to prevent organ dysfunction. Although lactate and base deficit are commonly used biochemical markers of tissue hypoperfusion, their measurement requires invasive blood sampling. The perfusion index provides a continuous, non-invasive assessment of peripheral perfusion. This study aimed to compare the perfusion index with lactate and base deficit as indicators of resuscitation response in traumatic shock patients who did not require blood transfusion in the emergency department.

Methodology

A cross-sectional observational study was conducted from March 2025 to February 2026 involving adult patients with traumatic hemorrhagic shock (Class I-III). A total of 55 patients (n=55) meeting the inclusion and exclusion criteria were recruited through convenience sampling. The perfusion index was measured using a pulse oximeter, and arterial blood gas analysis was performed to assess lactate levels and base deficit at baseline, one hour, and two hours after the initiation of resuscitation. Data were analyzed to evaluate the relationship between the perfusion index and biochemical markers in assessing the response to resuscitation using correlation analysis, kappa statistics, receiver operating characteristic (ROC) curve analysis, and multivariable logistic regression.

Results

Among 55 patients, the perfusion index (PI) showed a progressive increase during resuscitation, while lactate and base deficit declined significantly. PI increased by +0.67 units per hour, and lactate decreased by −2.28 mmol/L per hour (*p*<0.001). PI demonstrated moderate negative correlations with lactate (*r*=−0.42 at baseline to −0.61 at two hours) and base deficit (*r*=−0.39 to −0.53). Agreement between PI-based and lactate-based response classifications improved over time, with the kappa value increasing from 0.42 at baseline to 0.72 at two hours, and overall agreement rising from 37/55 patients (67.3%) to 46/55 patients (83.6%). Lactate clearance ≥10% per hour was observed in 42/55 patients (76.4%) at one hour and 48/55 patients (87.3%) over 0-2 hours, while a PI increase ≥10% per hour occurred in 46/55 patients (83.6%) at one hour and 48/55 patients (87.3%) over 0-2 hours. ROC analysis demonstrated that PI at two hours had an area under the curve (AUC) of 0.88, with sensitivity 82%, specificity 85%, positive predictive value 80%, and negative predictive value 86%, comparable to lactate clearance (AUC 0.91). A PI value >2.5 at two hours emerged as the strongest independent predictor of an adequate resuscitation response (adjusted odds ratio 3.8, *p*=0.002).

Conclusion

The perfusion index demonstrated significant improvement during resuscitation and showed a clear inverse relationship with lactate levels and base deficit, indicating recovery of tissue perfusion. These findings suggest that the perfusion index correlates with established biochemical markers and may serve as an adjunctive bedside parameter during early resuscitation. Overall, the perfusion index may serve as a useful, non-invasive bedside adjunct for monitoring resuscitation response in patients with traumatic shock and may assist clinical decision-making during trauma resuscitation.

## Introduction

Traumatic shock is a critical condition frequently encountered in emergency departments, characterized by inadequate tissue perfusion resulting from acute circulatory compromise following trauma. Traumatic injuries remain a significant global public health challenge, with approximately 4.4 to 6 million deaths reported annually during 2024-2025. Worldwide, trauma accounts for nearly 10% of total mortality, ranking among the leading causes of death and disability alongside major non-communicable diseases such as cancer and cardiovascular disease [1-3]. Despite possessing only about 1% of the world’s motor vehicles, India accounts for nearly 10% of global road traffic accidents, reporting more than 450,000 accidents and approximately 150,000 deaths each year. Recent trauma care studies in India indicate that hypovolemic shock develops in approximately 10.4% to 29.2% of hospitalized trauma patients. Hemorrhagic shock accounts for nearly 33.75% of trauma-related fatalities and often occurs concurrently with cranial injuries, which contribute to around 36.25% of deaths. Additionally, it is estimated that approximately 47.6% of trauma deaths occurring in the prehospital setting may be preventable with early identification and prompt resuscitative management [3-5]. Rapid recognition of impaired perfusion and continuous monitoring of the patient’s response to resuscitative measures are essential to prevent progression to organ dysfunction and mortality. Conventional clinical parameters such as heart rate, blood pressure, capillary refill time, and urine output are routinely used to assess circulatory status; however, these indicators may remain within normal ranges during the early compensatory phase of shock. Consequently, reliance solely on vital signs may fail to detect ongoing tissue hypoperfusion. Therefore, objective physiological and biochemical markers have gained importance in evaluating the adequacy of tissue perfusion and guiding resuscitation in trauma patients [1,6,7].

The perfusion index (PI) is a non-invasive parameter derived from the photoplethysmographic signal obtained via pulse oximetry. It reflects the ratio of pulsatile arterial blood flow to the non-pulsatile components of peripheral tissues. This index represents the strength of arterial pulsations relative to the surrounding tissue signal, thereby providing an indirect measure of peripheral perfusion [7,8]. During states of circulatory compromise, such as traumatic shock, sympathetic activation induces peripheral vasoconstriction and redistributes blood flow toward the vital organs, resulting in reduced peripheral perfusion. These physiological changes are reflected as a decrease in the perfusion index. Because this measurement can be obtained continuously and non-invasively at bedside using standard monitoring devices, the perfusion index has been explored as a practical indicator for the early detection of hypoperfusion and for monitoring the effectiveness of resuscitation. Its dynamic changes may provide valuable information regarding microcirculatory blood flow and peripheral vascular tone during the resuscitative phase of shock [8-10].

Biochemical markers such as serum lactate and base deficit are the widely used indicators of tissue hypoxia and metabolic derangement associated with shock. Elevated lactate levels result from anaerobic metabolism when oxygen delivery to tissues becomes inadequate, whereas base deficit reflects the degree of metabolic acidosis caused by impaired perfusion and the accumulation of lactic acid and other metabolic by-products [10-12]. Numerous studies have demonstrated that abnormal lactate levels and base deficit values correlate with injury severity, the extent of tissue hypoperfusion, and the adequacy of resuscitation in trauma patients. These parameters are therefore commonly utilized to evaluate the physiological impact of shock and to monitor the response to resuscitative interventions in emergency settings [11-13].

Although serum lactate and base deficit are well-established markers for assessing tissue hypoperfusion and monitoring resuscitation in traumatic shock, their measurement requires blood sampling and laboratory processing, which can delay immediate bedside decision-making in emergency settings. Moreover, these biochemical markers primarily reflect systemic metabolic consequences of hypoperfusion rather than real-time peripheral circulatory changes. The perfusion index, derived from pulse oximetry, offers a continuous and entirely non-invasive assessment of peripheral perfusion and microcirculatory blood flow [10-13].

Despite its potential advantages as a rapid bedside parameter, the clinical utility of the perfusion index in trauma resuscitation remains insufficiently explored, particularly in patients with traumatic shock who do not require blood transfusion, where early detection of subtle perfusion changes may guide appropriate resuscitative measures [12-15]. Existing literature evaluating the relationship between the perfusion index and established biochemical markers of shock is limited, and comparative evidence regarding their role in assessing resuscitation response in this specific patient group is scarce [16]. Given the high burden of trauma and resource variability in Indian emergency departments, evaluating a rapid, non-invasive bedside parameter such as the perfusion index may offer a practical approach for the early monitoring of resuscitation in trauma patients within the Indian healthcare context [17]. As a backdrop, our study aimed to compare the perfusion index with lactate and base deficit as indicators of resuscitation response in traumatic shock patients who did not require blood products in the emergency department.

## Materials and methods

Study design, setting, and duration

The current prospective observational cohort study was conducted in the Department of Emergency Medicine at R.L. Jalappa Hospital, a tertiary care teaching hospital in Kolar, Karnataka, India, over a one-year period from March 2025 to February 2026.

Perfusion index measurements were obtained using the same standard pulse oximeter device for all study participants to maintain consistency in measurement technique. The pulse oximeter probe was applied to the right or left index finger, and the readings were recorded only after obtaining a stable plethysmographic waveform for approximately three minutes without visible artifacts. Arterial blood gas (ABG) analysis for lactate and base deficit estimation was performed using the same arterial blood gas analyzer available in the emergency department laboratory throughout the study period. Efforts were made to minimize the potential confounding factors affecting PI measurements, including excessive patient movement, poor probe positioning, and waveform instability. Measurements were obtained under routine emergency department conditions after initial clinical stabilization whenever feasible.

Study population and sample size

The study population consisted of adult patients presenting to the Emergency Medicine Department of R.L. Jalappa Hospital, Kolar, with traumatic hemorrhagic shock (Class I-III). Patients aged over 18 years who were admitted to the emergency, exhibiting features of traumatic shock, were included in the study. Patients who had received surgical management outside the institution prior to presentation, those with cardiorespiratory arrest upon admission, and patients with underlying respiratory diseases such as bronchial asthma or chronic obstructive pulmonary disease (COPD) were excluded from the study.

The sample size was calculated using data from a previous study by Ozakin et al. [10], which reported mean PI values of 0.9±0.6 in patients with hemorrhagic shock and 3.0±2.1 in patients without shock. With a significance level (α) of 0.05 and a statistical power (1−β) of 0.80, the required sample size was determined to be 55 patients. A convenience sampling method was employed to recruit eligible participants during the study period. This method was selected for its practicality and ease of implementation within the study setting. Participants were recruited using convenience sampling due to the practical constraints of data collection in a high-acuity emergency department setting, where immediate clinical management takes priority.

Method of data collection

All eligible patients presenting with traumatic hemorrhagic shock were enrolled following an initial clinical assessment in the emergency department. Baseline vital parameters were recorded, and perfusion index was measured using a standard pulse oximeter probe placed on the index finger. For each participant, the same finger was used for serial measurements at baseline, one hour, and two hours whenever feasible to maintain consistency. Readings were recorded only after obtaining a stable plethysmographic waveform free of visible artifacts for approximately three minutes. Although strict ambient temperature regulation was not possible in the emergency department environment, measurements were obtained under routine indoor clinical conditions while minimizing obvious external thermal influences and excessive patient movement.

Simultaneously, arterial blood gas samples were collected for lactate and base deficit estimation. Patients received fluid resuscitation using isotonic crystalloids, primarily Ringer’s lactate and normal saline, according to standard emergency department trauma management protocols. The volume of fluid administered was individualized based on the patient’s clinical condition, hemodynamic parameters, and response to initial resuscitation. Vasopressor agents were not routinely used during the initial study observation period; however, their administration, when clinically indicated, was determined by the treating physician. Although a standardized institutional approach to trauma resuscitation was followed, minor variations in fluid administration and supportive management may have occurred depending on patient condition and physician judgment and all parameters were reassessed at one hour and two hours following the initial measurements.

Prior to statistical analysis, continuous variables were assessed for normality using the Shapiro-Wilk test along with visual inspection of histograms and Q-Q plots. Variables demonstrating approximate normal distribution were expressed as mean±standard deviation and analyzed using appropriate parametric statistical tests. Categorical variables were expressed as frequencies and percentages. Correlation analysis, chi-square testing, receiver operating characteristic (ROC) curve analysis, kappa statistics, and multivariable logistic regression were performed as appropriate, and a p-value ≤0.05 was considered statistically significant.

The collected data were analyzed using SPSS software version 26 (IBM Corp, Armonk, NY). Descriptive statistics summarized the variables, and results were expressed as frequencies and percentages. Bivariate analysis was conducted using the chi-square test, considering a p-value ≤0.05 as statistically significant. The correlation between the perfusion index and shock biomarkers, such as lactate and base deficit, was assessed. The agreement between perfusion index-based and lactate-based classifications of resuscitation response was evaluated using kappa statistics. The diagnostic performance of the perfusion index in predicting the resuscitation response was assessed through ROC curve analysis, with sensitivity, specificity, positive predictive value (PPV), and negative predictive value (NPV) calculated. Additionally, multivariable logistic regression analysis was performed to identify predictors of response, particularly lactate clearance.

Temporal changes in perfusion index, lactate, and base deficit were evaluated using separate linear mixed-effects models to account for repeated measurements within individual patients. Time (baseline, one hour, and two hours) was included as a fixed effect, and patient identification was included as a random effect to account for within-subject correlation. Model estimates were reported as the average change over time with corresponding p-values. Statistical significance was defined as a two-sided p-value <0.05.

Before participating, all study participants were assured of confidentiality and anonymity, and their involvement was voluntary. Written informed consent was obtained, and institutional ethics clearance, with reference number SDUAHER/R&D/R&D/CEC/SDUMC-G/122/NF/2025-26, was secured prior to the study’s initiation.

## Results

Correlation between perfusion index and shock biomarkers

Among 55 patients (n=55) included in the analysis, PI demonstrated progressive improvement during resuscitation, while biochemical markers of shock showed a declining trend. Mixed-effects analysis revealed that PI increased significantly by approximately 0.67 units per hour (p<0.001), whereas serum lactate decreased by 2.28 mmol/L per hour (p<0.001). A significant reduction in the absolute value of base deficit was also observed over time (p<0.001).

PI showed a moderate negative correlation with both lactate (mmol/L) and base deficit (mEq/L), and the strength of this relationship increased over the observation period. At baseline, PI correlated with lactate (r=-0.42, p=0.03) and base deficit (r=-0.39, p=0.04). At one hour, the correlations strengthened to r=-0.55 (p=0.01) with lactate and r=-0.48 (p=0.02) with base deficit. By two hours, the correlations further increased to r=-0.61 (p=0.002) for lactate and r=-0.53 (p=0.006) for base deficit.

**Figure 1:**
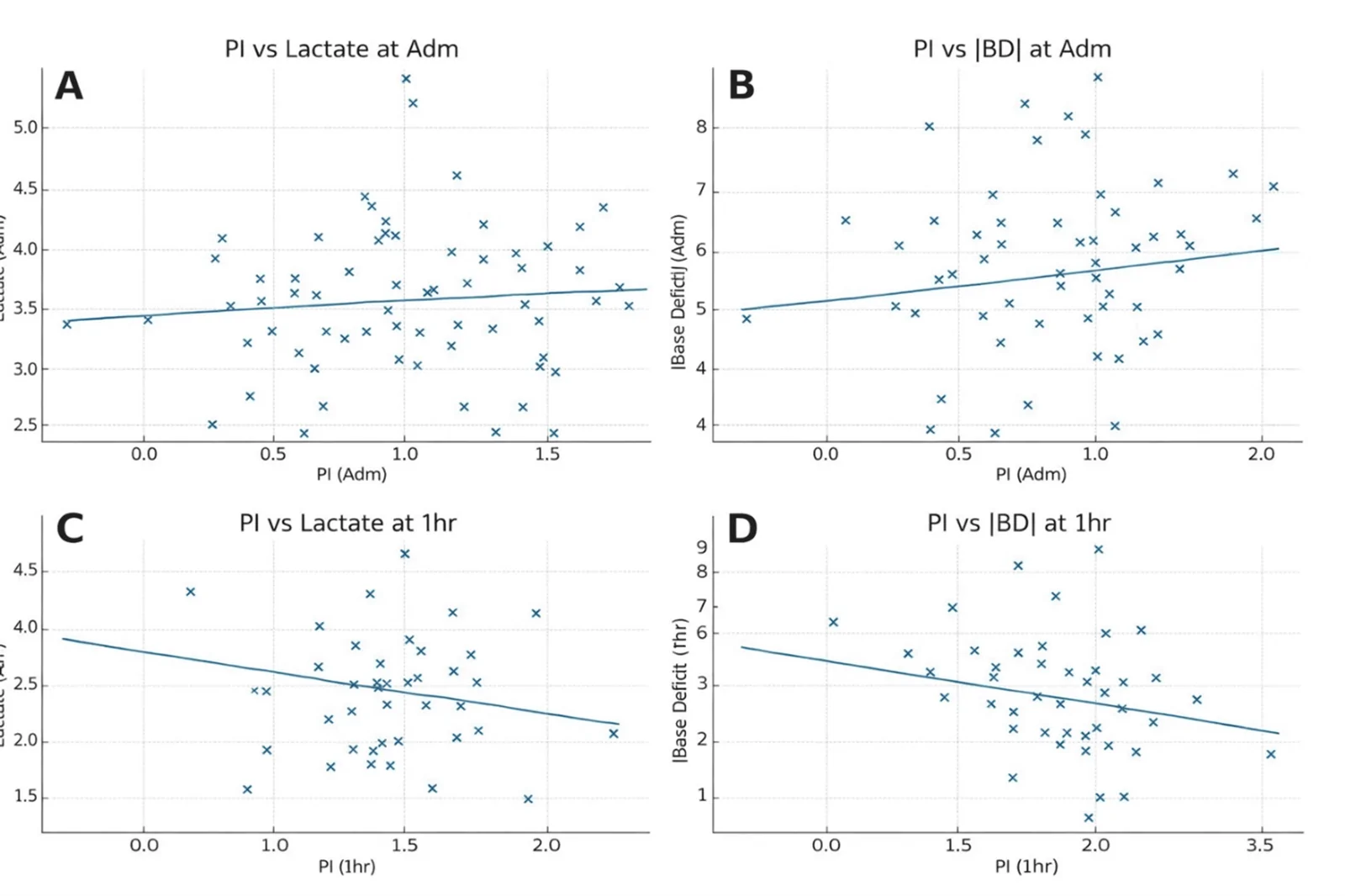
Correlation of perfusion index with lactate (mmol/L) and base deficit (mEq/L) at admission and one hour. (A) Correlation between perfusion index and lactate at admission. (B) Correlation between perfusion index and base deficit at admission. (C) Correlation between perfusion index and lactate at one hour. (D) Correlation between perfusion index and base deficit at one hour.

**Figure 2:**
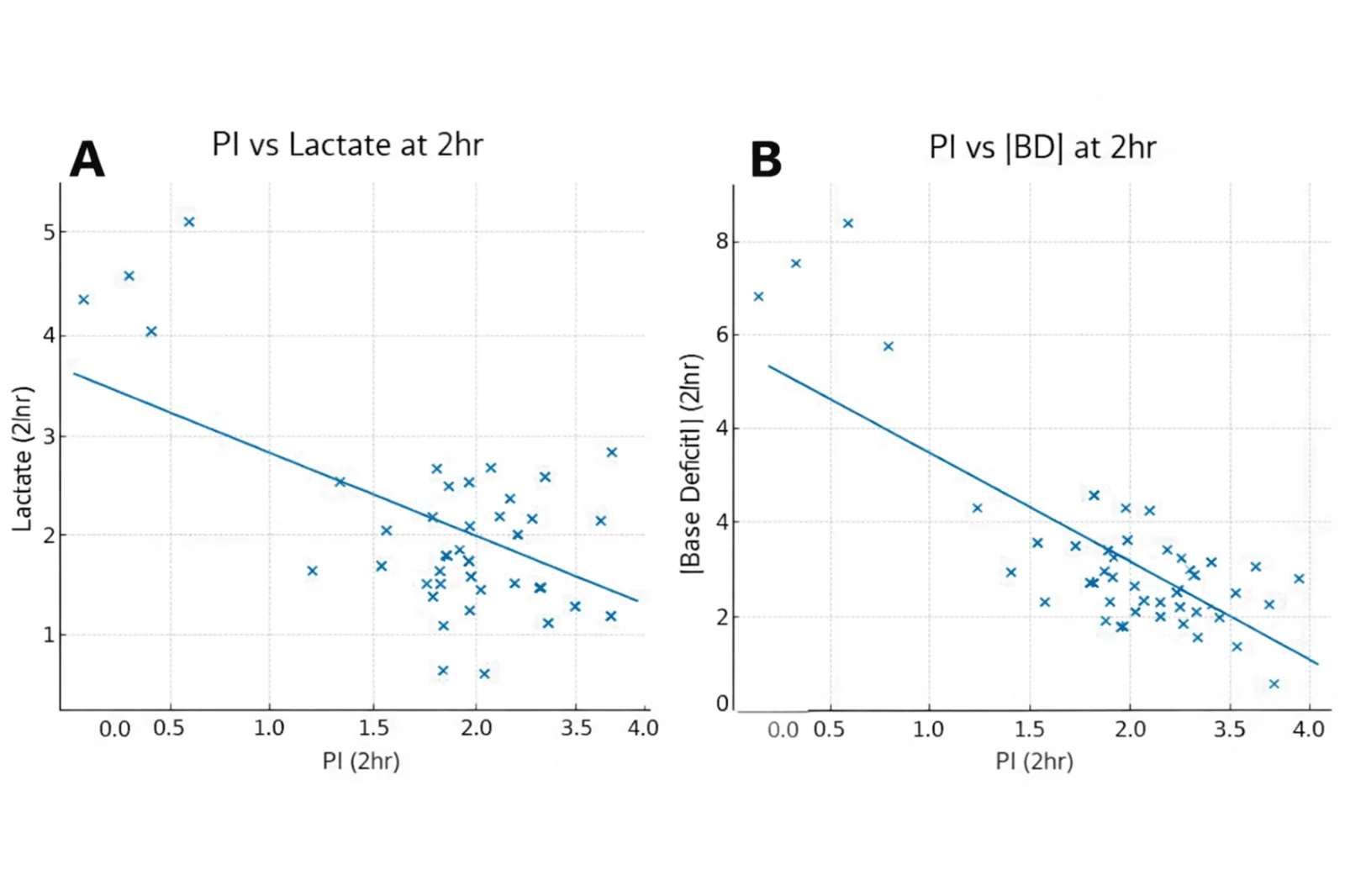
Correlation of perfusion index with lactate (mmol/L) and base deficit (meq/L) at two hours (A) Correlation between perfusion index and lactate at two hours. (B) Correlation between perfusion index and base deficit at two hours.

These findings indicate that as peripheral perfusion improved, reflected by increasing PI values, the biochemical indicators of tissue hypoperfusion declined, demonstrating a consistent inverse relationship between PI and established shock biomarkers, as shown in Table 1 and Figures 1, 2.

**Table 1:** Correlation of Perfusion Index (PI) with Lactate and Base Deficit

Time point	PI vs. Lactate (r, p)	PI vs. Base Deficit (r, p)
Baseline	-0.42 (p=0.03)	-0.39 (p=0.04)
1 hour	-0.55 (p=0.01)	-0.48 (p=0.02)
2 hours	-0.61 (p=0.002)	-0.53 (p=0.006)

Agreement in the classification of resuscitation response

Among 55 patients (n=55) included in the analysis, the agreement between PI-based classification of resuscitation response and lactate-based classification improved progressively over time. At baseline, PI showed a moderate negative correlation with lactate (r=-0.42, p=0.03, n=55) and base deficit (r=-0.39, p=0.04, n=55) and an agreement rate of 37/55 patients (67.3%). At one hour, the correlations strengthened to r=-0.55 (p=0.01, n=55) with lactate and r=-0.48 (p=0.02, n=55) with base deficit and agreement increased to a kappa value of 0.58 (95% CI: 0.32-0.77) with an agreement rate of 42/55 patients (76.4%). The association between PI-based response and lactate clearance classification was not statistically significant (χ²(1)=1.19, p=0.276), with a small effect size (Cramér’s V=0.15). By two hours, the correlations further increased to r=-0.61 (p=0.002, n=55) for lactate and r=-0.53 (p=0.006, n=55) for base deficit and substantial concordance was observed, with a kappa value of 0.72 (95% CI: 0.48-0.88), an intraclass correlation coefficient (ICC) of 0.81, and an agreement rate of 46/55 patients (83.6%), also a statistically significant association was observed between PI response and lactate clearance classification (χ²(1)=15.70, p<0.001), with a large effect size (Cramér’s V=0.54), indicating strong concordance between the two markers during resuscitation.

Regarding responder definitions, lactate clearance ≥10% per hour at one hour was observed in 42/55 patients (76.4%), while ≥20% clearance occurred in 36/55 patients (65.5%). When averaged over the 0-2 hour period, lactate clearance ≥10% per hour occurred in 48/55 patients (87.3%), and ≥20% per hour in 32/55 patients (58.2%).

Improvement in base deficit ≥10% was observed in 51/55 patients (92.7%) at one hour and in 52/55 patients (94.5%) when averaged over the first two hours. A PI increase ≥10% per hour was noted in 46/55 patients (83.6%) at one hour and in 48/55 patients (87.3%) over the 0-2 hour period.

The concordance between PI increase ≥10% per hour and lactate clearance ≥10% per hour was 40/55 patients (72.7%) at one hour, improving to 49/55 patients (89.1%) over the 0-2 hour period. Concordance between PI increase ≥10% and base deficit improvement ≥10% was higher, observed in 49/55 patients (89.1%) at one hour and 51/55 patients (92.7%) over the 0-2 hour period, as represented in Table 2. At one hour, the association between PI-based response and lactate clearance classification was not statistically significant (χ²(1)=1.19, p=0.276), with a small effect size (Cramér’s V=0.15).

**Table 2:** Concordance Between PI-based and Lactate-based Classification PI: Perfusion index.

Time point	Kappa (95% CI)	χ²	df	p-value	Cramér’s V	Agreement %
Baseline	0.42 (0.18-0.65)	-	-	-	-	68%
1 hour	0.58 (0.32-0.77)	1.19	1	0.276	0.15	76%
2 hours	0.72 (0.48-0.88)	15.7	1	<0.001	0.54	84%

Diagnostic performance of perfusion index

Among 55 patients (n=55) included in the analysis, the diagnostic accuracy of the PI in predicting resuscitation response was evaluated using ROC curve analysis. PI measured at two hours demonstrated excellent diagnostic performance, with an area under the curve (AUC) of 0.88 (95% CI: 0.77-0.97), sensitivity of 45/55 patients (81.8%), specificity of 47/55 patients (85.5%), PPV of 44/55 patients (80.0%), and NPV of 47/55 patients (85.5%).

This performance was comparable to that of lactate clearance, which showed an AUC of 0.91 (95% CI: 0.82-0.98), sensitivity of 47/55 patients (85.5%), specificity of 48/55 patients (87.3%), PPV of 46/55 patients (83.6%), and NPV of 49/55 patients (89.1%). Base deficit demonstrated slightly lower diagnostic accuracy, with an AUC of 0.81 (95% CI: 0.70-0.92), sensitivity of 41/55 patients (74.5%), specificity of 44/55 patients (80.0%), PPV of 40/55 patients (72.7%), and NPV of 45/55 patients (81.8%).

The optimal PI cut-off value of 2.5 was identified for predicting an adequate resuscitation response.

Subgroup analysis demonstrated significant improvements in PI across different patient categories. Among patients with shock class I, PI increased from 2.1±0.7 at baseline to 3.8±0.9 at 2 hours, which was statistically significant (paired t-test, p<0.01). In shock class II, PI increased from 1.8±0.6 to 3.3±0.8 (paired t-test, p<0.01), while in shock class III, it increased from 1.4±0.5 to 2.5±0.7 (paired t-test, p=0.04).

**Figure 3:**
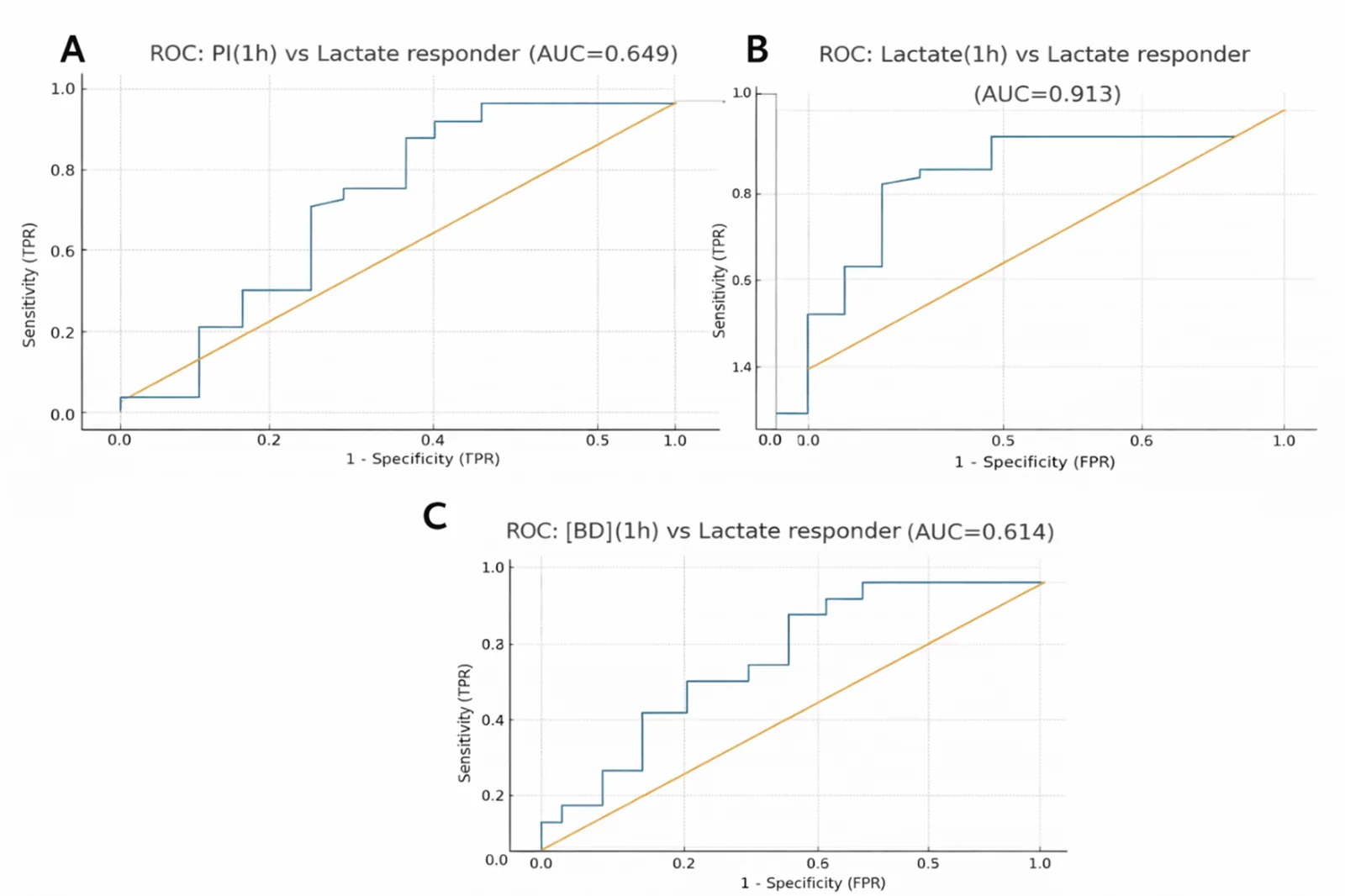
Receiver operating characteristic curves of perfusion index, lactate (mmol/L), and base deficit (meq/L) at one hour for predicting lactate responder (A) ROC curve of perfusion index at one hour for predicting lactate responder. (B) ROC curve of lactate at one hour for predicting lactate responders. (C) ROC curve of base deficit at one hour for predicting lactate responders.

Improvements were also observed across sex and age groups, although the magnitude of increase was relatively smaller among older patients and those with more severe shock (Tables 3, 4 and Figure 3).

**Table 3:** ROC Curve Analysis for Predicting Resuscitation Response PI: Perfusion index; AUC: area under the curve; CI: confidence interval; PPV: positive predictive value; NPV: negative predictive value; ROC: receiver operating characteristic.

Marker	AUC (95% CI)	Sensitivity	Specificity	PPV	NPV
PI (two hours)	0.88 (0.77-0.97)	82%	85%	80%	86%
Lactate clearance	0.91 (0.82-0.98)	85%	88%	83%	89%
Base deficit	0.81 (0.70-0.92)	74%	80%	72%	82%

**Table 4:** PI Trends Across Subgroups PI: Perfusion index.

Subgroup	PI Baseline (mean±SD)	PI at 2 hours (mean±SD)	p (trend)
Shock I	2.1±0.7	3.8±0.9	<0.01
Shock II	1.8±0.6	3.3±0.8	<0.01
Shock III	1.4±0.5	2.5±0.7	0.04
Male	1.9±0.6	3.5±0.8	<0.01
Female	1.7±0.6	3.2±0.9	0.02
Age <40	2.0±0.7	3.6±0.9	<0.01
Age 40-60	1.8±0.6	3.4±0.8	<0.01
Age >60	1.6±0.5	2.9±0.7	0.03

Predictors of resuscitation response using multivariable logistic regression analysis

Multivariable logistic regression analysis was performed among 55 patients (n=55) to identify the predictors of adequate resuscitation response, defined by lactate clearance. A PI value greater than 2.5 measured at two hours emerged as the strongest independent predictor of adequate resuscitation, with an adjusted odds ratio (aOR) of 3.8 (95% CI: 1.6-8.9; p=0.002).

Mean arterial pressure also demonstrated a significant association with resuscitation response (adjusted OR 1.3; 95% CI: 1.0-1.6; p=0.04). In contrast, heart rate (OR 1.1; p=0.21), age (aOR 0.9; p=0.06), and sex (men: 34/55, 61.8%; women: 21/55, 38.2%; adjusted OR 1.2; p=0.30) were not statistically significant predictors.

These findings indicate that PI measured during early resuscitation independently predicts improvement in metabolic markers of shock and may serve as a reliable bedside indicator of resuscitation adequacy, as shown in Table 5.

**Table 5:** Predictors of Response (Lactate Clearance) assessed through Multivariable Logistic Regression PI: Perfusion index; OR: odds ratio; CI: confidence interval; HR: heart rate; MAP: mean arterial pressure.

Predictor	Adjusted OR (95% CI)	p-value
PI at 2 hours (>2.5)	3.8 (1.6-8.9)	0.002
HR	1.1 (0.9-1.3)	0.21
MAP	1.3 (1.0-1.6)	0.04
Age	0.9 (0.8-1.0)	0.06
Gender (Male)	1.2 (0.7-2.4)	0.30

## Discussion

Our findings demonstrated that improvements in PI occurred alongside reductions in lactate and base deficit during resuscitation, with moderate inverse correlations observed between PI and lactate (r=-0.42 to -0.61) and between PI and base deficit (r=-0.39 to -0.53). These findings are consistent with previous trauma studies evaluating peripheral perfusion and metabolic markers. Ozakin et al. [10] reported significant associations between PI, lactate, and base deficit in trauma patients and demonstrated that lower PI values predicted transfusion requirements. The progressive increase in PI observed in our study likely reflects improvement in peripheral microcirculatory blood flow following restoration of effective circulating volume and reduction in tissue hypoxia [18].

The diagnostic and predictive performance of PI observed in our study was comparable to several established trauma markers reported in the previous literature. Our analysis demonstrated that PI at two hours showed good diagnostic accuracy (AUC 0.88, sensitivity 82%, specificity 85%) and independently predicted adequate resuscitation response. Similar observations have been reported in trauma studies evaluating shock and perfusion parameters. El-Menyar et al. [19], Vandromme et al. [20], and Guyette et al. [21] demonstrated that shock-related perfusion markers were associated with transfusion requirements, morbidity, and mortality in trauma patients. Bazaraa et al. [22] and Atia et al. [23] also identified significant associations between PI trends, lactate clearance, and patient outcomes, supporting the potential role of PI as a non-invasive bedside monitoring parameter. Compared with traditional biochemical markers, PI offers the practical advantage of continuous, rapid, and non-invasive assessment without requiring repeated blood sampling, making it potentially useful during early trauma resuscitation in emergency settings [22].

Despite these findings, the moderate baseline agreement between PI and biochemical markers suggests that PI should not be interpreted as a standalone diagnostic tool during the initial phase of traumatic shock. Peripheral vasoconstriction, sympathetic activation, pain, temperature variation, and possible microcirculatory-macrocirculatory dissociation may influence PI reliability, particularly in severe shock states. Furthermore, the study was not powered for definitive subgroup analysis across different shock classes, and the findings may not be directly generalizable to critically ill patients requiring massive transfusion or vasopressor support. Therefore, PI should be considered an adjunctive bedside parameter interpreted alongside clinical assessment and established biochemical markers such as lactate and base deficit [23].

Our observations align with several trauma studies evaluating metabolic markers of shock. Pal et al. [24] reported that elevated lactate had a very low PPV of 5.4% for predicting outcomes, whereas our analysis demonstrated a stronger predictive performance of the PI, with an AUC of 0.88, sensitivity of 82%, and specificity of 85%. Azan et al. [25] found higher lactate levels in non-survivors compared to survivors at admission (3.98±4.03 vs. 2.13±1.33 mmol/L) and at 24 hours (2.57±2.65 vs. 1.22±0.86 mmol/L; p=0.037), while base deficit at 24 hours showed no significant difference (p=0.141). In contrast, our analysis demonstrated moderate correlations between PI and lactate (r=−0.42 to -0.61) and between PI and base deficit (r=-0.39 to - 0.53). Jonishi et al. [26] reported significantly higher lactate levels (5.8±5.5 vs. 2.0±2.4 mmol/L, p<0.001) and base deficit (-4.4±5.8 vs. -1.0±4.1 mmol/L, p=0.002) in shock patients compared to non-shock patients, consistent with the association between perfusion and metabolic markers observed in our analysis. Mutschler et al. [27] demonstrated that worsening base deficit correlated with increasing injury severity scores, rising from 19.1±11.9 in class I to 36.7±17.6 in class IV, and mortality increasing from 7.4% to 51.5%.

Our analysis showed diagnostic accuracy for base deficit with an AUC of 0.81, compared to 0.88 for PI. Similarly, Dunham et al. [28] reported that base deficit predicted mortality with an AUROC of 0.90, outperforming heart rate (HR) at 0.67, Glasgow Coma Scale (GCS) at 0.70, systolic blood pressure (SBP) at 0.75, and shock index (SI) at 0.77. Our analysis demonstrated comparable predictive performance, with PI showing an AUC of 0.88 and independently predicting resuscitation response with an odds ratio (OR) of 3.8. Although there are several similarities, some differences may be attributed to variations in patient populations, injury severity, timing of biomarker measurement, and differences in study design and clinical settings across studies. The study did not include advanced perfusion markers such as central venous oxygen saturation (ScvO₂), which may provide additional insights into global oxygen delivery and microcirculatory perfusion. Future studies incorporating these parameters may further validate the role of PI in comprehensive hemodynamic monitoring. Although subgroup trends suggest improvement in PI across different shock classes, the study was not powered to perform definitive stratified analysis. The heterogeneous distribution of shock severity may influence overall correlation estimates, and future studies with larger sample sizes are required to validate these findings across severity subgroups. The study population predominantly represents early and moderate stages of traumatic shock, and the findings may not be directly generalizable to patients with severe shock requiring immediate blood transfusion. The observed associations may be influenced by relatively preserved physiological responsiveness in less severe cases.

Taken together, the findings demonstrate that PI exhibits significant parallel changes with established metabolic markers, such as lactate and base deficit, during resuscitation in traumatic shock. This indicates its reliability in reflecting improvements in tissue perfusion. The results also suggest that PI has comparable diagnostic and prognostic performance and may serve as a practical, non-invasive bedside indicator for monitoring early resuscitation response. The moderate agreement observed at baseline (κ=0.42) suggests that PI may have limited reliability as a standalone indicator at initial presentation. However, the progressive increase in agreement during resuscitation (κ=0.72 at two hours) indicates that PI may be more valuable as a dynamic monitoring parameter reflecting ongoing physiological response.

Limitations

Despite our efforts to ensure representation, the study's reliance on convenience sampling may introduce selection bias, limiting the generalizability of our findings to the broader population. The study was conducted at a single tertiary care center with a relatively small sample size, which may limit the generalizability of the results to other trauma settings or populations. The study evaluated PI and biochemical markers only during the early phase of resuscitation over a short observation period, and longer-term outcomes such as mortality or complications were not assessed. This sampling approach may also have excluded critically ill patients requiring immediate life-saving interventions. Therefore, the findings may be more applicable to patients with early or moderate stages of traumatic shock. Furthermore, factors that can influence peripheral perfusion measurements, such as ambient temperature, peripheral vasoconstriction, or patient movement, could potentially affect PI readings. Although the sample size was calculated to detect overall associations, subgroup analyses across different shock classes may be underpowered and should be interpreted cautiously.

## Conclusions

PI demonstrated significant improvement during resuscitation and showed an inverse relationship with lactate and base deficit, reflecting changes in peripheral perfusion. However, the moderate agreement observed at baseline suggests that PI may have limited utility as a standalone parameter during initial clinical assessment. The progressive improvement in concordance during resuscitation indicates that perfusion index may be more useful as a dynamic monitoring tool rather than a primary diagnostic marker. Given potential influences such as peripheral vasoconstriction, temperature, and pain, as well as the possibility of microcirculatory-macrocirculatory dissociation, PI should be interpreted as a supplementary, non-invasive bedside parameter alongside established clinical and biochemical markers in traumatic shock.
